# Urothelial Cancer Associated 1 (UCA1) and miR-193 Are Two Non-coding RNAs Involved in Trophoblast Fusion and Placental Diseases

**DOI:** 10.3389/fcell.2021.633937

**Published:** 2021-05-13

**Authors:** Clara Apicella, Camino S. M. Ruano, Sébastien Jacques, Géraldine Gascoin, Céline Méhats, Daniel Vaiman, Francisco Miralles

**Affiliations:** ^1^Institut Cochin, Université de Paris, U1016 INSERM, UMR 8104, CNRS, Paris, France; ^2^Unité Mixte de Recherche MITOVASC, Équipe Mitolab, CNRS 6015, INSERM U1083, Université d’Angers, Angers, France; ^3^Réanimation et Médecine Néonatales, Centre Hospitalier Universitaire, Angers, France

**Keywords:** trophoblast, placenta, preeclampsia, intra uterine growth restriction, syncytialisation, non-coding RNAs

## Abstract

A bioinformatics screen for non-coding genes was performed from microarrays analyzing on the one hand trophoblast fusion in the BeWo cell model, and on the other hand, placental diseases (preeclampsia and Intra-Uterine Growth Restriction). Intersecting the deregulated genes allowed to identify two miRNA (mir193b and miR365a) and one long non-coding RNA (UCA1) that are pivotal for trophoblast fusion, and deregulated in placental diseases. We show that miR-193b is a hub for the down-regulation of 135 cell targets mainly involved in cell cycle progression and energy usage/nutrient transport. UCA1 was explored by siRNA knock-down in the BeWo cell model. We show that its down-regulation is associated with the deregulation of important trophoblast physiology genes, involved in differentiation, proliferation, oxidative stress, vacuolization, membrane repair and endocrine production. Overall, UCA1 knockdown leads to an incomplete gene expression profile modification of trophoblast cells when they are induced to fuse into syncytiotrophoblast. Then we performed the same type of analysis in cells overexpressing one of the two major isoforms of the STOX1 transcription factor, STOX1A and STOX1B (associated previously to impaired trophoblast fusion). We could show that when STOX1B is abundant, the effects of UCA1 down-regulation on forskolin response are alleviated.

## Introduction

In humans, abnormal placental development is associated with two major pregnancy diseases: preeclampsia (PE) and intrauterine growth restriction (IUGR).

PE occurs in a range of 2–5% pregnancies, and it is characterized by hypertension and proteinuria, surging from the mid-gestation at the earliest (Steegers et al., 2010). Despite a certain degree of heterogeneity in its pathogenesis, a consensus exists that abnormal placentation or placenta development could be at the origin of the disease. Notably, placental ischemia would cause intermittent hypoxia, oxidative stress, cell death, and the release to the maternal circulation of anti-angiogenic factors and debris that promote inflammation and a systemic endothelial dysfunction (Rana et al., 2019). In some cases, the disease poses a real threat to the survival of the mother requiring the delivery of the feto-placental unit. Thus, PE is one of the major causes of premature births (before 37 completed weeks of pregnancy), with their cortege of neonate complications (Goldenberg et al., 2008). The symptoms of the disease disappear after delivery. However, epidemiological studies have shown that the women who have suffered a preeclamptic pregnancy have an increased risk of developing a cardiovascular disease (CVD) later in life (Newstead et al., 2007; Brouwers et al., 2018), as well as other diseases affecting strongly vascularized tissues, such as the brain (Basit et al., 2018).

IUGR refers to a somehow loosely defined condition in which the unborn baby is smaller than expected for his or her gestational age (Nardozza et al., 2017). IUGR babies typically have an estimated weight that falls below that of 90% of unborn babies of the same gestational age. In addition, IUGR babies are sometimes born prematurely. Babies with IUGR are at increased risk of health problems before, and after birth. These problems include low oxygen levels while in the womb and high levels of distress during labor and delivery. In the long term, IUGR increases the risk of developing a metabolic disease such as type 2 diabetes and CVD (Darendeliler, 2019).

In about one third of the cases, PE is complicated with IUGR, suggesting that there could be an overlap in the etiology of both diseases. The considerable similarity in histopathology and gene expression in the placentas has been recently reported between both diseases (Awamleh et al., 2019; Gibbs et al., 2019; Medina-Bastidas et al., 2020).

In the human placenta, the maternal blood is in direct contact with a continuous multinucleated layer, the syncytiotrophoblast (STB). This polarized interface releases hormones and mediates the exchange of nutrients, gases and waste between mother and the developing fetus (Turco and Moffett, 2019). The STB is mitotically inactive, formation and constant renewal of the syncytium depends on the underlying mononuclear cytotrophoblasts (CTB). Throughout gestation, CTBs proliferate, differentiate and eventually fuse with the STB via cell-syncytial fusion. This process is balanced by a concomitant release of apoptotic material as syncytial knots from the STB to the maternal circulation. Hence, the process of syncytialization is critical to the integrity of the STB and in maintaining the essential functions of the placenta. Several *in vitro* and *in vivo* studies have demonstrated a close, if not a causal, relationship between structural/functional deficiency of the syncytium and the development of PE and IUGR (Guller et al., 2008; Roland et al., 2016; Costa, 2016).

Genome-wide transcriptomic and epigenomic studies have greatly contributed to the understanding of the molecular mechanisms involved in either normal or pathological placenta development. Thus, numerous studies have revealed altered placental expression of various genes in PE and IUGR (Cox et al., 2015; Deyssenroth et al., 2017; Chabrun et al., 2019; Majewska et al., 2019; Benny et al., 2020). A particular category concerns those genes encoding for non-protein coding RNAs (ncRNAs). Classes of ncRNAs include transfer RNAs (tRNAs), ribosomal RNAs (rRNAs), small RNAs, such as microRNAs (miRNAs), siRNAs, piRNAs, snoRNAs, snRNAs, exRNAs, scaRNAs and the long ncRNAs (Hombach and Kretz, 2016). The ncRNAs display a great variety of mechanisms of action including: post-transcriptional gene regulation through controlling processes like protein synthesis, RNA maturation, transport and decay, but also, transcriptional gene regulation through the modification of chromatin structure (Fernandes et al., 2019). They are an important basis of epigenetic regulation in the human placenta, in normal and pathological situations (Hayder et al., 2018; Apicella et al., 2019). Structurally different ncRNAs engage diverse mechanisms that lead to different regulatory outcomes. The discovery of the diversity of functions played by the ncRNAs in the cell physiology has boosted the exploration of their role in placental development, physiology and pathology.

The central role of the STB in the physiology of the placenta, suggests that deregulation of ncRNAs specifically required for its formation and/or maintenance could be potentially involved in placental diseases. Here we combined two microarray analyses, one carried out on the classical fusogenic trophoblast model BeWo (under the accession number GSE148088) (Kudo et al., 2004; Ramos et al., 2008; Orendi et al., 2010; Shankar et al., 2015; Zheng et al., 2016), and one carried out on total human placentas with normal controls, PE and IUGR placentas (under the accession number E-MTAB-9416). A cross-analysis was carried out with a drastic filtering in order to identify ncRNA that are associated to disease (in the placentas) and to fusion (induced by forskolin treatment in the BeWo cells), in parallel.

**FIGURE 1:**
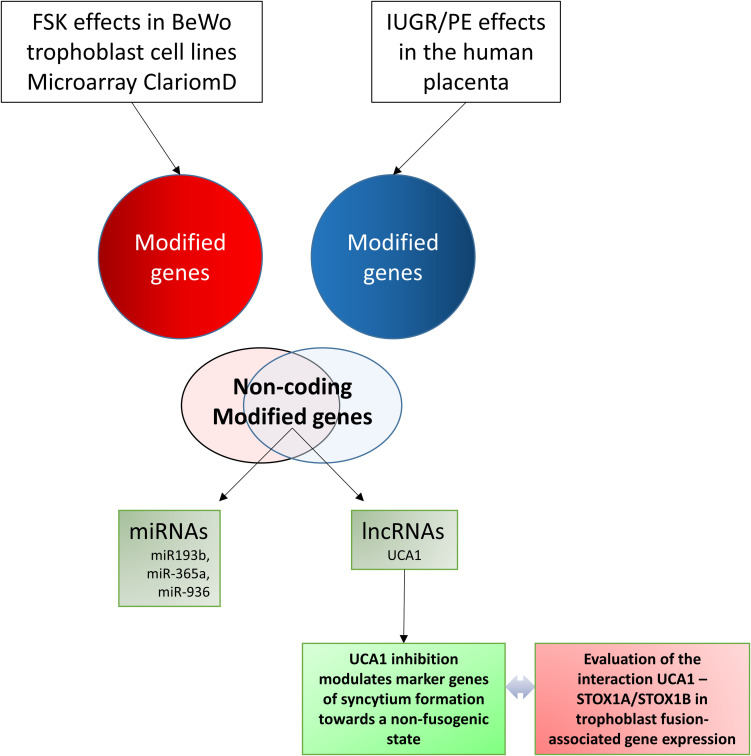
Chart of the study, explaining the pivotal aim: connecting non-protein coding genes involved in trophoblast fusion with placental genes altered in human placental pathologies (Preeclampsia and Intra-Uterine Growth Restriction).

This cross-analysis allowed the identification of a small subset of ncRNAs which are consistently modified both during fusion of trophoblast cells, and in the pathological placentas. We then carried on our analysis focusing upon the miRNA miR-193b (by a bioinformatic approach) and the lncRNA UCA1 (through knock-down (KD) experiments). In addition, we analyzed the effects of this KD in BeWo cells, overexpressing specifically one of the two major isoforms of the STOX1 transcription factor (STOX1A and STOX1B), previously identified as a key player in preeclampsia (George and Bidwell, 2013), and recently shown to modulate fusion through a specific equilibrium between its two isoforms (Vaiman and Miralles, 2016; Ducat et al., 2020). The chart of the present study is shown as Figure 1.

## Results

### Transcriptional Modifications in BeWo Cells Following Forskolin Treatment

**FIGURE 2:**
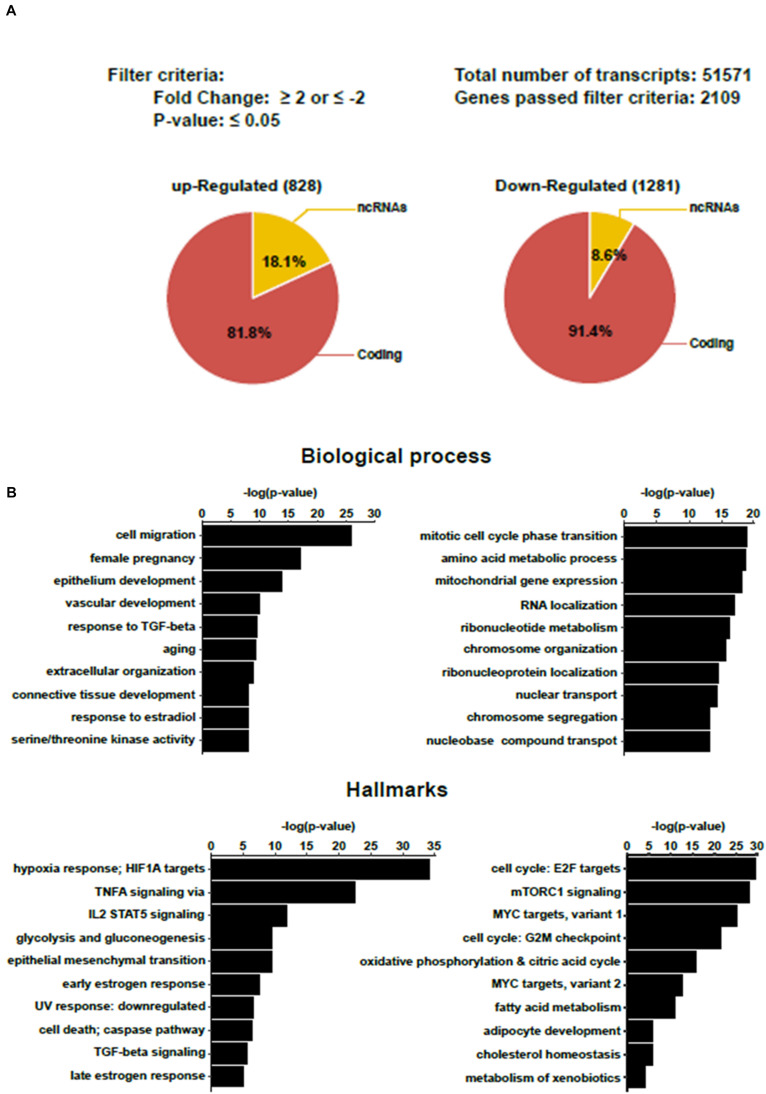
Transcriptomic analysis of BeWo cells after 72 h treatment with forskolin. **(A)** Summary of transcriptomic analysis. Genes with a fold change (FC) either ≤-2 or ≥2, and *P* ≤ 0.05 were considered differentially expressed in the BeWo cells treated with forskolin (BeWo-FSK) relative to the untreated cells (BeWo-CO). **(B)** Over Representation Analyis (ORA) showing principal Biological processes and Hallmarks enriched by the up-regulated (left) and down-regulated (right) DEGs in the BeWo-FSK compared to the BeWo-CO.

BeWo cells were cultured in the presence of 20 μM forskolin to induce cell fusion (BeWo-FSK). Control cells were grown with the vehicle, DMSO (BeWo-CO). After 72 h, the total RNA was extracted, and global gene-expression profiles were analyzed with microarrays. Comparison of BeWo-FSK relative to BeWo-CO detected 2109 genes differentially expressed (DEGs) with a fold change (FC) either ≤-2 or ≥2, and *P*-value ≤ 0.05 (Figure 2A and Supplementary Table 1). Of these, 828 genes were up-regulated and 1,281 down-regulated (Supplementary Table 1). Gene set enrichment analysis (GSEA), and over representation analysis (ORA) confirmed that our results were accurately consistent with previous reports having analyzed transcriptomic changes in BeWo cells after forskolin treatment (Supplementary Figure 1). In the BeWo-FSK we detected substantially increased expression of key markers of syncytialization such as CGA (*FC* = 10.1; *P* = 2.1 × 10^–11^), CGB1 (*FC* = 20.5; *P* = 2.81 × 10^–12^) or ERVFRD-1 (aka Syncytin2, *FC* = 10.6; *P* = 6.23 × 10^–12^). Up-regulated DEGs were associated with biological processes such as cell migration, vascular development, response to TGF-Beta and response to hypoxia. Down-regulated DEGs are mainly involved in cell cycle progression, amino-acid metabolism, and mitochondrial gene expression (Figure 2B). In terms of hallmarks present in the GSEA Broad database, inflammation and hypoxia pathways were particularly enriched in up-regulated genes, while cell cycle, nutrient sensing via mTORC1 pathway, were strongly enriched in down-regulated genes, suggesting a whole silencing of basic pathways of cell physiology and energy expenditure slow down, accompanying the differentiation of the trophoblast cells into a syncytial structure.

### Differentially Expressed Non-coding RNAs in Forskolin-Treated BeWo Cells

**FIGURE 3:**
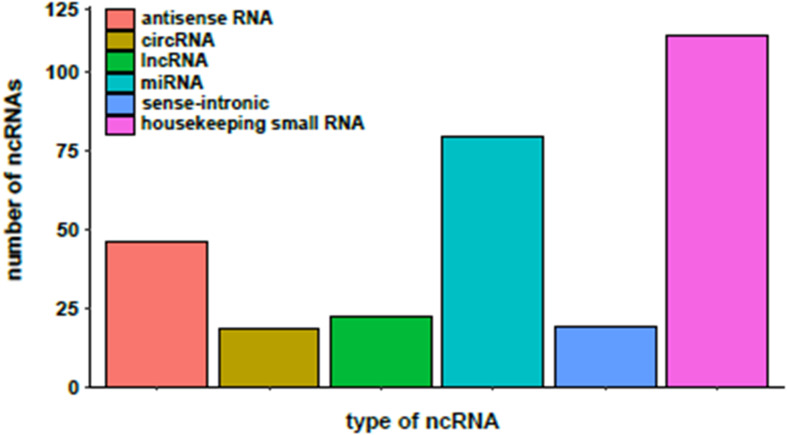
Distribution of the different categories of non-coding RNAs (ncRNAs) differentially expressed in the BeWo-FSK.

Three hundred and seven (307) of the DEGs detected in the BeWo-FSK relative to BeWo-CO, encode annotated ncRNAs. They belong to different categories including: sense-intronic RNA, antisense RNA, long non-coding RNA (lncRNA), circular (circRNA), microRNA (miRNA) and housekeeping ncRNAs (Y-RNAs, ribosomal RNAs, Small nucleolar RNAs and transfer RNAs) (Figure 3). The category of housekeeping ncRNAs was the most represented (111 genes). Nonetheless, we focused our study on the categories corresponding to regulatory ncRNAs. A selection of the most significantly modified regulatory ncRNAs detected following forskolin treatment in the BeWo cells is shown in Table 1. These include the lncRNAs, antisenseRNAs and miRNAs. The list of the totality of ncRNAs (classified by category) detected as differentially expressed is provided as Supplementary Table 2.

**TABLE 1:** Top 25 differentially expressed regulatory ncRNAs in the BeWo cells after 72 h of forskolin treatment.

**Gene symbol**	**ncRNA class**	**Fold change**	***P*-value**	**FDR *P*-value**
hsa-miR-147b	miRNA	28.94	8.33E-11	3.53E-07
LINC01511	lncRNA	14.88	8.43E-13	1.63E-08
hsa-miR-4632	miRNA	8.93	6.38E-09	7.80E-06
RP11-420L9.5	antisenseRNA	7.6	1.61E-08	1.48E-05
LINC01237	lncRNA	4.45	2.70E-07	1.00E-04
MYCNUT	lncRNA	4.31	3.72E-09	5.15E-06
LINC01164	lncRNA	3.98	1.37E-07	8.14E-05
UCA1	lncRNA	3.76	2.19E-06	7.00E-04
CTB-60B18.12	antisenseRNA	3.46	2.06E-06	7.00E-04
hsa-miR-6810	miRNA	3.36	3.13E-06	9.00E-04
hsa-miR-936	miRNA	3.35	5.99E-06	1.50E-03
SLC2A1-AS1	antisenseRNA	3.23	6.17E-06	1.50E-03
IL10RB-AS1	antisenseRNA	3.14	5.32E-06	1.30E-03
hsa-miR-193b	miRNA	2.91	8.00E-04	4.54E-02
hsa-miR-365a	miRNA	2.73	3.90E-06	1.10E-03
hsa-miR-6888	miRNA	2.54	8.24E-05	9.80E-03
hsa-miR-3941	miRNA	2.16	3.18E-05	5.10E-03
Hsa-miR-636	miRNA	−2.16	4.69E-05	6.70E-03
hsa-miR-301a	miRNA	−2.27	2.92E-05	4.90E-03
RP11-884K10.7	antisenseRNA	−2.84	4.00E-04	2.68E-02
COX10-AS1	antisenseRNA	−3.12	5.63E-05	7.60E-03
OLMALINC	lncRNA	−3.33	9.98E-05	1.12E-02
hsa-miR-1908	miRNA	−3.45	6.12E-07	3.00E-04
DLEU2	lncRNA	−3.75	8.74E-05	1.03E-02
hsa-miR-6758	miRNA	−4.31	8.16E-07	3.00E-04

### Identification of Regulatory ncRNAs Targets Reveals Their Potential Roles in Syncytialization

To investigate the role of these ncRNAs in the process of BeWo fusion we identified their putative targets (when known) using appropriate databases (miRBase, starBase v2.0. and DianaTools LncBase v.2). Since miRbase tends to provide a more limited list of putative target genes (∼10% of the others) for a given miRNA, and since these were generally largely covered in the other databases, this constituted the major basis for the establishment of our lists of targets. Next, we selected among these targets those which are indeed detected as DEGs in the BeWo-FSK relative to the BeWo-CO cells. These resulted in a list of 278 up-regulated and 572 down-regulated DEGs. Out of the deregulated DEG list (Supplementary Table 1), this represents 33.6% for the up-regulated and 44.6% for the down-regulated DEGs. Assuming a total of 50,000 genes including the non-protein coding ones, the expected proportions are 1.6 and 1.1%, respectively, thus we observed a significant enrichment of putative targets in both cases (χ^2^ contingency test, *p* < 10^–20^).

Hallmark enrichment analysis using the ncRNAs targeted-DEGs revealed that the most significantly impacted functions are related to the cell cycle progression for the down-regulated DEGs, while TNF-signaling and hypoxia response are the most enriched processes for the up-regulated DEGs. Strikingly, these enriched pathways are quite similar to those obtained with the total DEG gene set, suggesting that many DEGs contributing to the definition of the hallmarks are targeted by the ncRNAs.

### Network Analysis of ncRNAs and Their Targets Identifies miR-193b as a Hub

**FIGURE 4:**
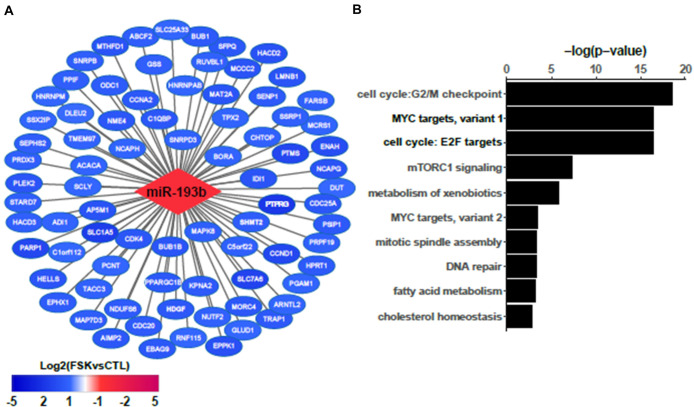
Network analysis identifies miR193b as a hub regulator **(A)** Most significantly down-regulated genes in BeWo-FSK targeted by miR193b. **(B)** Hallmarks enrichment analysis of the targets of miR193b deregulated in the BeWo-FSK relative to the BeWo-CO.

To further analyze the role of the differentially expressed ncRNAs in the process of BeWo fusion we used the Cytoscape tool to construct a regulatory network integrating these ncRNAs and their targets. This resulted in a network composed of 985 nodes (representing ncRNAs and targets) and 1,775 edges (representing interactions). Next, we submitted our network to topological analysis. MiR-193b was identified as the principal hub of our network, with 135 predicted targets (Figure 4A). Other lesser hubs corresponding to miR-16, miR-455, and miR-365 are presented as Supplementary Figure 2. Most of the targets of miR-193b were down-regulated (108 out of 135) in the BeWo-FSK relative to BeWo-CO, while miR-193b was significantly up-regulated (*FC* = 2.91; *P* = 0.0008). Hallmarks enrichment analysis shows that the down-regulated genes targeted by mir-193b are mainly involved in the control of cell cycle progression and energy usage/nutrient metabolism (Figure 4B).

### A Small Subset of ncRNAs Involved in Syncytialization Is Also Associated With Preeclampsia and Intrauterine Growth Restriction

To identify ncRNAs associated with syncytialization in BeWo which could be also involved in PE and/or IUGR we compared the list of ncRNAs identified in BeWo with those identified in a list from our study on total human placentas with normal controls, PE and IUGR placentas (E-MTAB-9416) and other published datasets (GSE114349, GSE114691, GSE75010, GSE93839, and GSE66273). Comparison of all differentially expressed ncRNAs, revealed that two miRNAs (miR-193b and miR-365a) and one lncRNA (UCA1) were found most consistently up-regulated in both PE and IUGR. Therefore, miR-193b is associated to trophoblast fusion, together with pathological placentation, suggesting its overall implication in normal placental function. A few additional miRNAs where also found simultaneously differentially expressed in preeclamptic placentas and in the BeWo-FSK (miR-936; miR-6886; miR-7110; miR-518A1; miR-4454, and miR-1283-2), but those were not studied further in the present paper.

**FIGURE 5:**
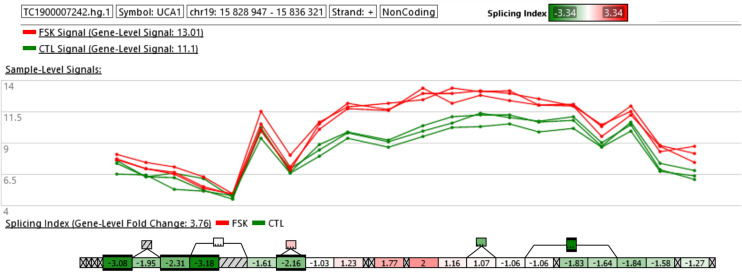
Exon level analysis of UCA1. The microarray ClariomD provides information on the local fluorescence level of a series of probe and allows to calculate a local splicing index, comparing BeWo cells treated with FSK vs. BeWo cells treated by the DMSO as a vehicle. Isoforms lacking the first portion of the full transcript are more strongly expressed in the BeWo-FSK comparatively to the BeWo-CO.

We next focused our attention on the lncRNA UCA1. This was justified by the following: (i) UCA1 is up-regulated in PE and IUGR but also in primary cultures of human syncytiotrophoblasts exposed for 24 h to 1% oxygen as compared to the same cells exposed to 20% oxygen (Table 2), (ii) also, a study coupling laser microdissection to isolate specific trophoblast subpopulations and microarray analysis, identified UCA1 as the most differentially expressed ncRNA in STBs isolated from the placentas of pregnancies with severe PE relative to controls (Gormley et al., 2017), (iii) *in situ* hybridization confirmed up-regulation of UCA1 in the STBs compartment of the placenta. Alternative splicing isoforms have been described for UCA1 (Xue et al., 2016). We found that UCA1 was increased in expression by 3.76-fold following FSK treatment. Exon level analysis of UCA1 (accessible through the ClariomD array used in this study) shows that probes located in the terminal part of the gene have a higher level of fluorescence in the forskolin-treated compared to the control cells (Figure 5). Thus, it shows that in the BeWo-FSK there is a specific increase in the production of the shorter isoforms (ENST00000600160.2, ENST00000589333.2) of UCA1, while the level of expression of the most complete isoform (ENST00000397381.4) is apparently unaffected.

**TABLE 2:** Genome-wide transcriptomic studies showing up-regulation of UCA1 either in preeclampsia or intrauterine growth retardation.

**Data set**	**Study**	**Fold change**	***P*-value**
E-MTAB-9416	IUGR vs. CO	8.91	3.20 × 10^–03^
GSE75010	PE vs. CO	2.04	2.81 × 10^–10^
GSE93839	PE STB vs. CO STB	5.93	1.55 × 10^–03^
GSE66273	PE vs. CO	6.19	3.33 × 10^–04^
GSE147776	IUGR vs. CO	2.01	3.58 × 10^–02^
GSE41331	CTB 1%O2 vs. 20%O2	1.86	1.61 × 10^–02^

### UCA1 siRNA Knockdown Lead to Altered Regulation of Genes Involved in Fusion Mechanisms in BeWo Cells

**FIGURE 6:**
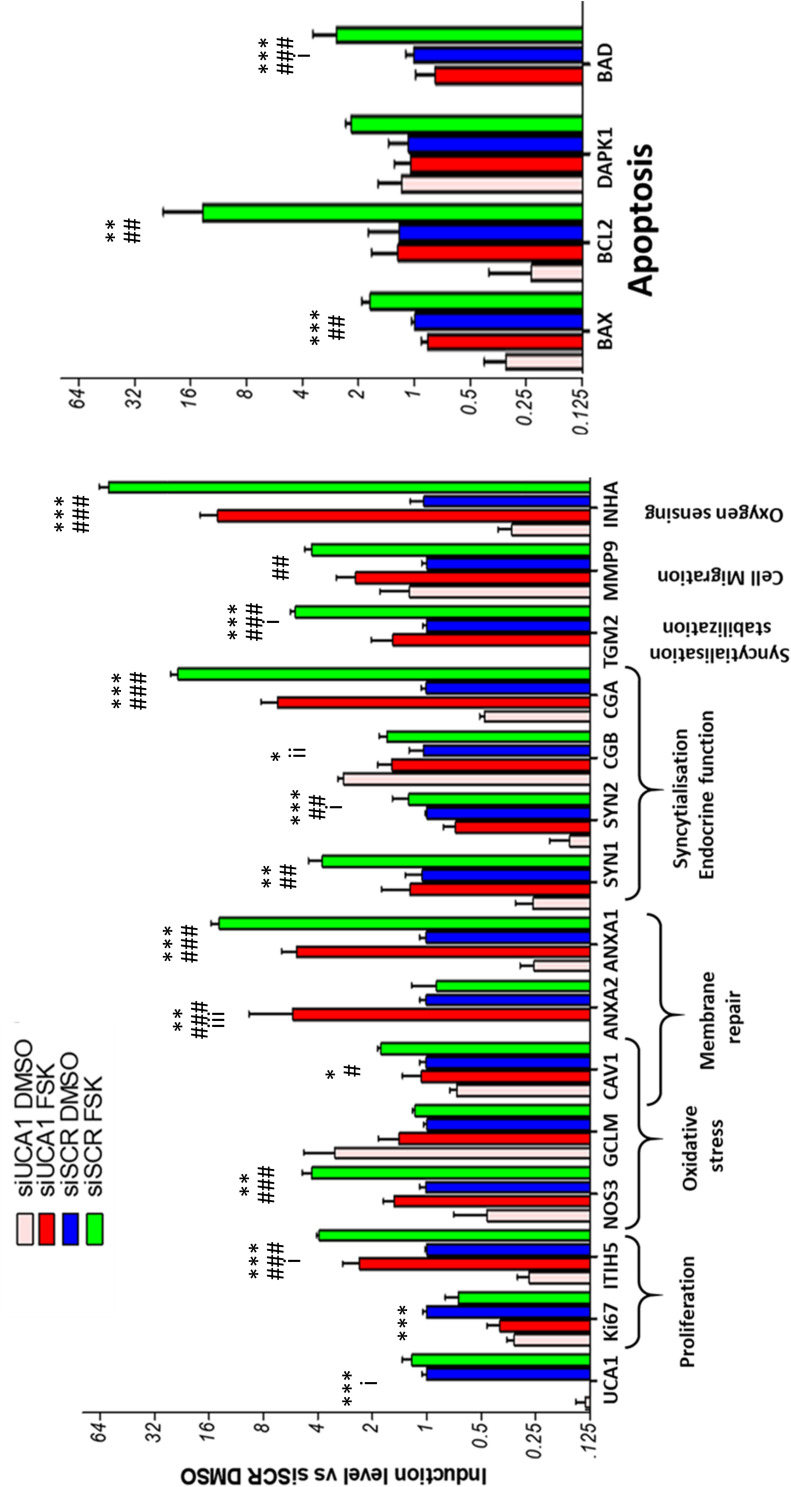
qRT-PCR analysis of the efficiency of UCA1 silencing in BeWo cells with or without forskolin treatment, and its effects on several markers of syncytialization. Genes involved in apoptosis are on the right panel. Stars (*) are marks of a significant effect of the siRNA against UCA1, hashes (#) are marks of significant effects of the forskolin-induced fusion, and (i) are marks of interaction effects (*, ^#^, i represent *p*-values below 0.05; **, ^##^, ii *p* < 0.01; ***, ^###^, iii*p* < 0.001).

**FIGURE 7:**
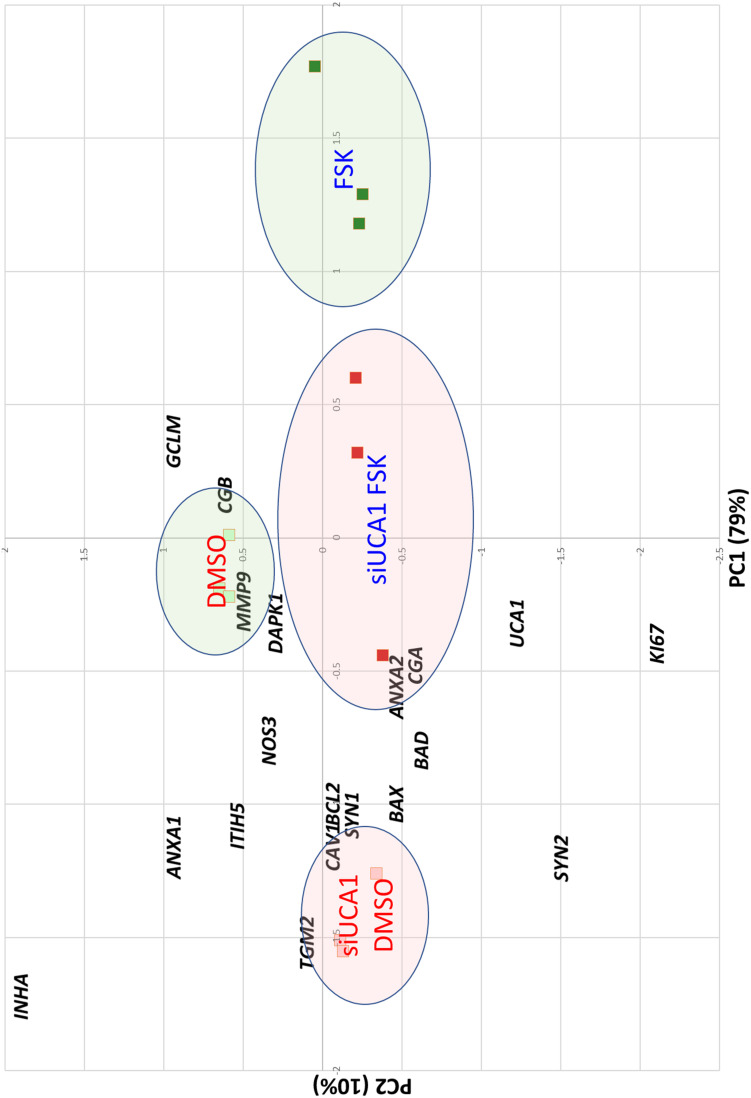
Principal Component Analysis of the BeWo cells treated or not with FSK and with or without the siRNA induced knockdown of UCA1. The squares are the samples analyzed projected on the two major principal components. The genes are projected as well on the factorial plan, to help for interpreting the axes. The horizontal axis is correlated with the cell differentiation induced by FSK. The second axis is rather correlated with cell proliferation. Circles are drawn to easily identify the different categories of treatment. Detailed interpretation is provided in the text.

The effect of the si-RNAs was evaluated by qRT-PCR. UCA1 levels were drastically affected (reduction ranging from 90 to 98% compared to the si-SCR, according to the experiment, Figure 6A). In a first characterization, we analyzed in BeWoC cells the expression of genes involved in cell proliferation (*ki67*, *ITIH5*), oxidative stress (*NOS3*, *GCLM* and *CAV1*-also involved in exosome physiology), membrane repair *(ANXA1, ANXA2*, *CAV1*), trophoblast fusion (*Syncytin1*, *Syncytin2*), endocrine differentiation of trophoblast (*CGA*/*CGB*), syncytiotrophoblast stabilization (*TGM2*) cell migration (*MMP9*), oxygen sensing (*INHA*), and apoptosis (*BAX*, *BCL2*, *DAPK1* and *BAD*). The KD of UCA1 led to significant alterations of almost all the genes involved in these pathways (Figure 6). A principal component analysis was carried out on the qPCR data and showed a clear separation of the cell replicates (Figure 7). The first axis (79% of the variation) is driven by the FSK treatment, while the second axis (10%) contrasts markers of differentiation vs. markers of proliferation. The analysis reveals that in terms of gene expression, the KD of UCA1 reduced the differences between FSK treated cells and control group in the center of the graph. This means that when levels of UCA1 are strongly reduced, the expression profile remains closer to that of untreated control cells. These observations suggest that UCA1 is important for a successful syncytialisation process.

A statistical test by two-ways ANOVA revealed significant modifications for most genes except GCLM and DAPK1 (Table 3 and Figure 6). The knockdown of UCA1 by itself affected all the genes, but MMP9. The FSK effect was significant in all the genes except UCA1, CGB, KI67 (in addition to GCLM and DAPK1). Finally, there was a significant interaction effect between the FSK treatment and the UCA1 KD in the case of UCA1, CGB, SYNCYTIN2, TGM2, ANXA2, ITIH5, and BAD. These interaction effects indicate a differential effect of the UCA1 KD according to the FSK treatment leading to cell fusion.

**TABLE 3:** Statistical analyses of individual gene effects following UCA1 knock-down ± FSK treatment (significant values are in red fonts).

**Overall tests of univariate models**			**Tests of univariate effects**		
**Y variable**	**F**	**Prob.**	**Source**	**F**	**Prob.**
UCA1	22.504	0.00029659	Si treatment	56.948	6.6307 × 10^–05^
			Fusion	4.288	0.07213567
			Si treatment*Fusion	6.275	0.03665572
CGB	8.208	0.00797657	Si treatment	10.373	0.01222583
			Fusion	0.283	0.60936093
			Si treatment*Fusion	13.967	0.00572781
CGA	127.930	4.243 × 10^–07^	Si treatment	43.849	0.00016558
			Fusion	336.346	8.0411 × 10^–08^
			Si treatment*Fusion	3.596	0.09452054
GCLM	2.521	0.13152052	Si treatment	4.143	0.07620217
			Fusion	0.975	0.3524659
			Si treatment*Fusion	2.445	0.15650114
NOS3	14.589	0.00131394	Si treatment	15.149	0.0045949
			Fusion	28.613	0.0006866
			Si treatment*Fusion	0.004	0.95003544
CAV1	5.155	0.02833346	Si treatment	7.939	0.02257829
			Fusion	7.147	0.02821257
			Si treatment*Fusion	0.379	0.55505375
KI67	13.163	0.00184323	Si treatment	34.327	0.00037894
			Fusion	0.891	0.37283458
			Si treatment*Fusion	4.272	0.07257943
SYN2	23.107	0.00027009	Si treatment	39.848	0.00022968
			Fusion	18.244	0.00271954
			Si treatment*Fusion	11.229	0.01006638
TGM2	51.718	1.3944 × 10^–05^	Si treatment	63.473	4.4987 × 10^–05^
			Fusion	84.869	1.5606 × 10^–05^
			Si treatment*Fusion	6.811	0.0311362
ANXA2	39.218	3.939 × 10^–05^	Si treatment	23.397	0.00129298
			Fusion	43.573	0.00016922
			Si treatment*Fusion	50.685	0.00010006
ITIH5	56.208	1.0168 × 10^–05^	Si treatment	37.157	0.00029085
			Fusion	125.667	3.5958 × 10^–06^
			Si treatment*Fusion	5.801	0.04260521
SYN1	10.209	0.00413556	Si treatment	15.939	0.00399228
			Fusion	14.682	0.00500579
			Si treatment*Fusion	0.005	0.94569912
ANXA1	96.898	1.2527 × 10^–06^	Si treatment	45.870	0.00014171
			Fusion	243.711	2.8262 × 10^–07^
			Si treatment*Fusion	1.114	0.32213654
MMP9	5.171	0.02810927	Si treatment	1.134	0.31791426
			Fusion	13.560	0.00619729
			Si treatment*Fusion	0.819	0.39182403
INHA	137.727	3.1788 × 10^–07^	Si treatment	40.502	0.00021729
			Fusion	372.037	5.4151 × 10^–08^
			Si treatment*Fusion	0.641	0.44650823
BAX	21.540	0.00034606	Si treatment	36.796	0.00030055
			Fusion	25.245	0.00102124
			Si treatment*Fusion	2.580	0.14686479
BCL2	8.774	0.00654947	Si treatment	13.427	0.00636233
			Fusion	12.786	0.00723255
			Si treatment*Fusion	0.110	0.74867557
DAPK1	2.339	0.14975061	Si treatment	2.106	0.18480454
			Fusion	2.027	0.1923598
			Si treatment*Fusion	2.884	0.12788132
BAD	32.681	7.7268 × 10^–05^	Si treatment	55.723	7.1629 × 10^–05^
			Fusion	36.270	0.0003154
			Si treatment*Fusion	6.050	0.03934242

### Interference Between UCA1 and the Trophoblast Differentiation Factor STOX1 in BeWo Cells

Previously, we have identified STOX1 as a major actor of the syncytialisation process in BeWo cells. More specifically, we showed that the short isoform of STOX1, STOX1B, antagonizes cell fusion when overexpressed and that disturbing the STOX1A/B balance mimics specific gene perturbations seen in PE CTBs in three well-characterized cell lines BeWoA, BeWoB and BeWoC, the first two overexpressing STOX1A and STOX1B, 20–30- and 6-fold, respectively (Ducat et al., 2020). In the present paper, analyzing exon-level microarrays, we show now for the first time that STOX1 isoforms are differentially spliced when the BeWo cells fuse under forskolin (Supplementary Figure 3), strengthening the idea that the two isoforms are associated with different pivotal stages of trophoblast differentiation.

**FIGURE 8:**
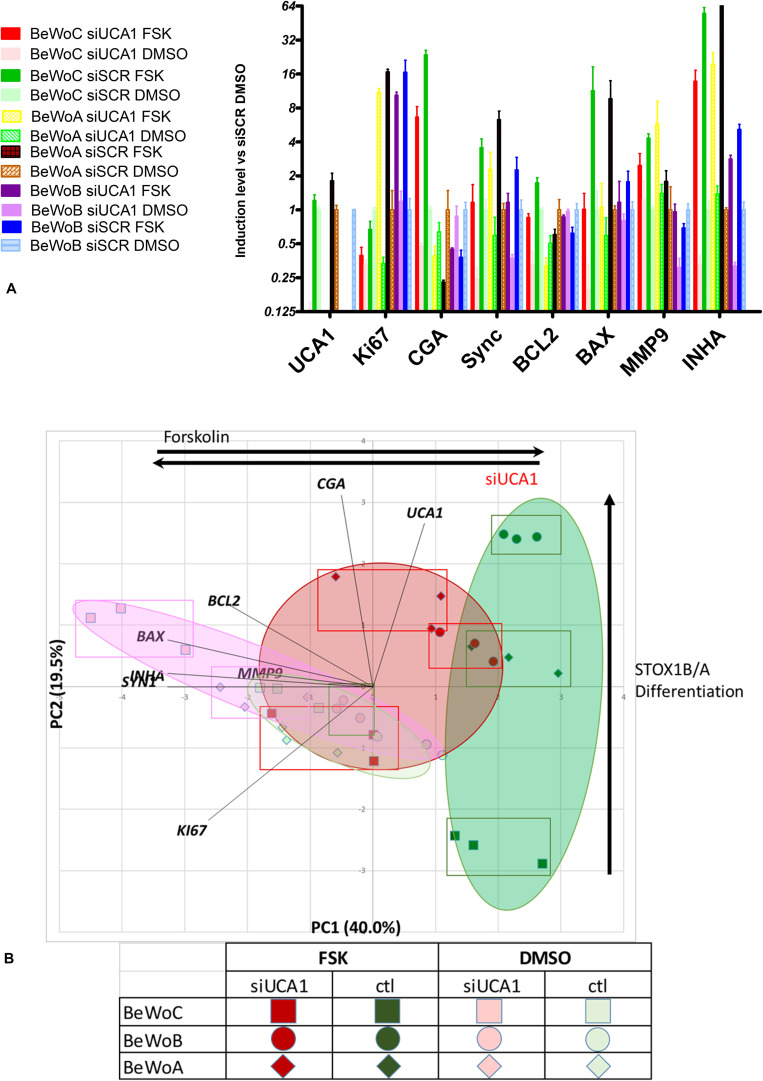
**(A)** qRT-PCR analysis of the efficiency of UCA1 silencing in controls BeWoC and BeWo overexpressing either STOX1A or STOX1B, with or without forskolin treatment, and its effects on several markers of syncytialization. **(B)** Principal Component Analysis of the BeWo cells [controls BeWoC (circles) and overexpressing either STOX1A (lozenges) or STOX1B (circles)]. The genes are represented in black bold characters. The rectangles show the position of the different replicates. Red color marks UCA1 KD, while green are control cases. Dark colors mark FSK treatment. The interpretation of the axes is marked by black arrows.

To explore the putative role of UCA1 in the syncytialization in normal and pathological conditions, we silenced this lncRNA using a specific small interfering RNA (si-RNA) in BeWo cells lines permanently transfected either with an empty plasmid (encoding G418 resistance), or with the same plasmid encoding for the expression of either STOX1A or STOX1B (These cells are called BeWoC, BeWoA or BeWoB, respectively). The cells were transfected with the si-UCA1 or a scrambled siRNA, si-SCR, used as control. We analyzed by qPCR a panel of 8 genes (including UCA1), The choice of these 8 genes was motivated by the fact that strong correlations exist between the 19 genes analyzed in the first experiment. The chosen genes are representative of the different pathways analyzed (proliferation-KI67, oxidative stress-INHA, trophoblast differentiation-SYN1, apoptosis-BCL2, BAX, invasion-MMP9, endocrine differentiation-CGA). The results are presented as a histogram (Figure 8A), with statistical values presented in Supplementary Table 3 (ANOVA), and as a PCA analysis, where the two first axes account for 40 and 19.5% of the variance (Figure 8B). BeWoC cells (represented by squares) harbor the same profile with the 8 genes from the

previous analysis: in the condition where the KD of UCA1 is combined with FSK treatment, the expression profile is close to the one of BeWoC cells without FSK and not treated with the siUCA1. Overall, the FSK effects were all oriented in the same direction of the first axis (clear to dark colors), which can thus be interpreted as a mark of FSK-induced trophoblast differentiation. A strong difference Between BeWoA, BeWoB and the control BeWoC comes from the variation along the second axis, showing that in this case, STOX1-overexpressing cells in the presence of forskolin are positioned with increasing abcissae, suggesting that this axis correlate with STOX1-driven differentiation in the presence of forskolin. When UCA1 is KD, the effect of STOX1 are less pronounced on the second axis (red squares, lozenges-BeWoA and circles-BeWoB). In the absence of FSK the STOX effects are less obvious and the dots are all in the middle of the graph.

Interestingly, the gene expression profiles are very peculiar in BeWoB cells under FSK treatment when analyzing UCA1 effects. On the first axis, UCA1 KD is not able to modify strongly the x-axis in these cells (from ∼2.3 to 1.5, Δ = 0.8), while in control BeWoC, the modification on the x-axis ranges from ∼1.9 to −0.5, Δ = 2.4, and in BeWoA from ∼2.2 to 0.5, Δ = 1.7. The effect on gene differentiation was thus less reduced in BeWoB cells, comforting the idea that BeWoB overexpression and UCA1 KD act similarly but not synergistically against trophoblast fusion. This may be associated to the fact that STOX1B overexpression is associated with deficits of syncytialization (Ducat et al., 2020).

## Discussion

In the placenta, the STB acts as a barrier between the mother and fetus, and functions in gas exchange, nutrient, waste transport and hormone production. The STB is mitotically inactive and is formed by the constant cell-cell fusion of the underlying mononuclear CTBs. STB fragments are continuously shed into the maternal circulation. Thus, maintenance of the STB requires a finely regulated turnover. Excessive or restricted CTB-STB fusion may lead to PE, IUGR, and implantation failure (Gauster et al., 2009).

Although derived from human choriocarcinoma, the BeWo cells, display structural and physiological features of human primary trophoblast (Burres and Cass, 1986; Ramos et al., 2008; Orendi et al., 2010) and have been largely used as a model to study the process of trophoblasts fusion induced by forskolin treatment (Chen et al., 2008; Zhou et al., 2013; Wang et al., 2014). Several transcriptomic studies have helped to identify important genes involved in trophoblast fusion (Kudo et al., 2004; Depoix et al., 2011; Shankar et al., 2015; Zheng et al., 2016). However, the majority of these studies have focused on the role of protein-coding genes. The role that ncRNAs could have in the trophoblast fusion remains to be explored.

Here, we have conducted a microarray transcriptomic analysis of BeWo cells under forskolin treatment (BeWo-FSK) and focused our analysis on the differentially expressed ncRNAs relative to controls (BeWo-CO). We identified a number of ncRNAs (antisense-RNAs, lncRNAs, miRNAs) which might be involved in the process of syncytialization *in vivo*. We have generated a network displaying the putative regulatory interactions among the differentially expressed ncRNAs and differentially expressed genes (DEGs) in the BeWo-FSK relative to the BeWo-CO. The analysis of this network shows that the majority of ncRNAs targets are involved in cell proliferation and metabolism. Topological analysis of the network identified miR193b as a principal hub of the network. Hallmarks enrichment analysis shows that most targets of miR193b are down-regulated genes involved in cell cycle progression such as CCND1, CCNA2, or BUB1B. Previous studies have shown that miR193b acts as tumor suppressor by repressing cell proliferation (Mazzu et al., 2017; Zhang et al., 2017; Bhayadia et al., 2018). In the context of placental development, it has been reported that mir193b-3p overexpression significantly decreases the migration and invasion of the trophoblast cells (HTR-8/SVneo) by targeting the 3′UTR of TGF-beta2 (Zhou et al., 2016). The miR193b has been found consistently up-regulated in PE and IUGR placentas in our study and others (Ishibashi et al., 2012; Xu et al., 2014; Zhou et al., 2016; Awamleh et al., 2019). This miRNA is thought to contribute to these pathologies because of its inhibitory effect on the migration and invasion of trophoblasts. Here we show that miR193b is also involved in the process of syncytialization. A prior, and key step in syncytialisation, is the acquisition of fusion competence, which requires the CTB to exit the cell cycle (Lu et al., 2017). Therefore, by targeting genes involved in the control of cell cycle progression, miR193b could play a pivotal role in this crucial step of the process of syncytialisation. Overexpression of miR193b in PE could negatively impact placental development by accelerating CTB-STB fusion, thus leading to a premature depletion of the pool of CTBs necessary to ensure the constant renewal of the STB. Alternatively, increased miR193b expression in PE could reflect a mechanism seeking to compensate an excessive apoptotic shedding of the syncytium by facilitating the entering of CTB into the fusion process.

Since a dysfunctional syncytium could be at the origin of placental diseases, we systematically searched for lncRNAs involved in the CTBs fusion that were deregulated in placentas from PE or IUGR. An exhaustive literature search, as well and the reanalysis of datasets available in the GEO Database resulted in the identification of three lncRNAs consistently up-regulated in PE and IUGR and involved in syncytialization: miR193b, miR365a and UCA1.

UCA1 (Urothelial Cancer Associated 1), is a lncRNA initially identified in a bladder cancer cell line (Wang et al., 2006). The involvement of lncRNAs in placental diseases has been previously described for the HELLP syndrome, a serious complication of preeclampsia (van Dijk et al., 2012). In the case of UCA1 high expression has previously been reported in different types of cancer. UCA1 promotes cell proliferation, tumor progression, migration and drug resistance. UCA1 mediates the transcriptional regulation at an epigenetic level by interaction with chromatin modifiers (EZH2, CTCF, YAP,…), by direct regulation via chromatin looping and/or by sponging miRNAs (Neve et al., 2018). The oncogenic functions of UCA1 have been extensively studied, but its role in development and differentiation remains unknown. A recent study, using the HTR-8/SVneo and JAR trophoblast cells suggests that UCA1 could inhibit trophoblast cell invasion and proliferation by down-regulating JAK2 (Liu et al., 2020). Knockdown of UCA1 in these cells suppressed the apoptotic rate and accelerated cell proliferation. Increased expression of UCA1 in PE had been reported previously and confirmed by our study (Liu et al., 2020). In addition, increased expression of UCA1, specifically in the STB of preeclamptic placentas, has been reported (Gormley et al., 2017). Thus, these data suggest that similar to miR193b, UCA1 might contribute in driving CTBs toward syncytialisation by inhibiting the genes involved in cell proliferation. The increased expression of UCA1 in the preeclamptic syncytium could reflect an increase in the turnover of the syncytium, to compensate for an increased apoptotic rate (Sharp et al., 2010; Fogarty et al., 2013). However, suppression of UCA1 in the HTR-8/SVneo and JAR cells decreased their apoptotic rates, indicating that UCA1 could also be involved in the induction of apoptosis (Liu et al., 2020). To explore more deeply the role that UCA1 might play in the process of syncytialization, we silenced its expression in the BeWo cells (BeWo-CO and BeWo-FSK) using a specific small interfering RNA (siRNA). Our results show that silencing UCA1 results in the downregulation of proliferation, attenuation of the expression of several markers of syncytialization, and most significantly downregulation of the expression of the antiapoptotic marker BCL2. As mentioned above in the HTR-8/SVneo and JAR cells, the Knockdown of UCA1, suppressed the apoptotic rate and accelerated cell proliferation (Liu et al., 2020). Thus, suppression of UCA1 in BeWo cells seems to have a different impact concerning the proliferation and apoptosis rates. These differences could be attributed to the fact that these cell lines are akin to different types of CTBs or either to particularities linked to the tumoral transformation process undergone by these cells. Nevertheless, our results on the BeWo cells are consistent with other studies indicating that in many cell types, UCA1 stimulates proliferation and inhibits apoptosis (Jun et al., 2018; Liu et al., 2018; Chen et al., 2019; Li et al., 2019; Wang et al., 2019). One limit of our study is that we did not carry out cell visualization experiments of the fusion; we can nevertheless assume that the alterations of syncytialisation marks that we observe here will likely be associated to phenotypic effects, that will have to be further assessed in future works.

Having in hand a model of altered fusion by STOX1A (BeWoA) or STOX1B (BeWoB) overexpression we attempted to evaluate the effect of UCA1 KD in these specific models. The overexpression of STOX1B (inhibiting fusion) led to more limited gene alterations than the other cells when forskolin was added in the Knock down of UCA1, suggesting that the alterations of gene expression induced by STOX1B overexpression render the UCA1 down-regulation alterations less visible.

Interestingly, hypoxia which plays a central role in the development of PE is known to induce UCA1 expression through HIF1A in both cancer cell lines and primary cultures of STB (Yuen et al., 2013; Xue et al., 2014; Zhu et al., 2019; Wang et al., 2020). In addition, it has been shown that UCA1 inhibits ischemia/reperfusion-induced apoptosis in cardiomyocytes (Chen et al., 2019; Wang et al., 2020). Given that ischemia/reperfusion is a known hallmark of severe PE and knowing that UCA1 has been detected as overexpressed specifically in the STB of severe preeclamptic patients (Gormley et al., 2017), it is tempting to speculate that UCA1 overexpression could reflect a protective mechanism aimed to attenuate apoptosis. However, in other cell types UCA1 has proved to exert pleiotropic effects, thus it might be involved in many other aspects of the trophoblast physiology. Although the BeWo cells are a good model to study the cell fusion process they are transformed carcinoma-like cells missing many trophoblast functions. Therefore, a more in-depth analysis of the functions of UCA1 in the placenta requires more physiological models such as primary trophoblast cultures, or placental organocultures. Another direction for future work is the assessment of the specific effects of the short isoforms of UCA1 that appear to be specifically modified when fusion occur. To note, we have recently demonstrated that alternative splicing is a general feature of placental disease, affecting hundreds of genes (Ruano et al., 2021). This could be achieved by lentiviral transformation of the cell lines, or even primary cultures with this short isoform. Finally, to understand better how UCA1 is actually functioning is an interesting challenge for further studies.

## Methods

### Cell Culture

BeWo cell lines were cultivated in F12 medium (Life Technologies) supplemented with 10% fetal bovine serum (FBS) and 1% penicillin/streptomycin in 6-cm diameter plates, up to 60% confluence and with 50 μg/ml of geneticin G-418. The generation of the BeWoA (overexpressing constitutively STOX1A), BeWoB (overexpressing constitutively STOX1B) and BeWoC cell lines is described in detail in Ducat et al. (2020). The concentration of forskolin chosen for this study was based on preliminary studies (Ducat et al., 2020). At the end of the treatment, total RNA was extracted as previously described (Ducat et al., 2016). The siRNA against UCA1 was Ambion silencer select provided by ThermoFisher scientific. After plating in 12-well plates (1 ml) at 70% confluency, the cells were transfected the nest morning using RNAiMAx transfection reagent (Invitrogen). Each well was transfected with 200 μl of Optimem^TM^ with 1 μl of RNAiMax and 0.5 μl of siRNA or si scrambled at 5 pmol/μl, following the manufacturer’s protocol. To induce syncytialization the cells were treated 1 day later with 20 μM forskolin or vehicle (DMSO) for 72 h.

### Microarray Assay

One hundred ng of RNA per sample were analyzed using the ClariomD (Affymetrix) microarray assay. Library preparation, hybridization and data acquisition were performed by GENOM‘IC platform according to manufacturer‘s instructions. Gene and exon level expressions were processed and extracted from the ClariomD microarray using the Transcriptomic Analysis Console (TAC) provided by Affymetrix.

### Quantitative Reverse Transcribed-PCR

Five hundred nanograms of total RNA were reverse transcribed with MMLV using the Invitrogen kit and random primers. qPCR was carried out under standard conditions in a LightCycler480 (Roche) in 96 well plates as previously described (Ducat et al., 2020), with a Sybrgreen kit from BioLine (Meridian Bioscience). In the case of UCA1, the analysis was carried out using a TaqMan probe and the Roche LightCycler^®^ TaqMan^®^ Master. The PPIA gene (cyclophilin) was used as reporter in all experiment, since we have shown previously an excellent stability of this gene in trophoblast cells. All the cell qPCR experiments were carried out three to four independent times, and every time in triplicates. Primers for the different genes are listed as Supplementary Table 4.

### Functional Annotation of the Differentially Expressed Genes (DEGs)

For the functional annotation of the DEGs we performed Over-representation analysis (ORA) using the WebGestalt^1^ bioinformatics resource (Liao et al., 2019). Databases interrogated include: Gene Ontology (GO), Kyoto Encyclopedia of Genes and Genomes (KEGG), and Hallmarks. The significance of the detected enrichments was calculated using the Benjamini and Hochberg multiple test adjustment.

### Gene Set Enrichment Analysis (GSEA)

GSEA was conducted using GSEA software from the Broad Institute^2^. The BeWo fusion gene set was generated using the top up-regulated and down-regulated genes after 72 h forskolin treatment reported by Shankar et al. (2015). We used as input the gene expression matrix generated by the Transcriptomic Analysis Console (Affymetrix) including all samples and replicates. The permutation value was set as 1,000. *P*-values were corrected for multiple testing and the cutoff for significant enrichment corresponds to an FDR < 0.25.

### Prediction of ncRNAs Targets

To identify targets for the differentially expressed ncRNAs in the BeWo-FSK relative to BeWo-Co cells we used *ad hoc* databases. These include miRBase^3^, starBase v2.0.^4^ and the DianaTools LncBase v.2^5^.

### ncRNAs Regulatory Network

The ncRNAs and corresponding differentially expressed targets were used to generate a regulatory network. The network was constructed, visualized and analyzed using the Cytoscape 3.2.1 software^6^ and its complementary applications (Shannon et al., 2003). The centrality parameters of the network were analyzed using the Cytoscape application NetworkAnalyzer (Shannon et al., 2003). Two topological parameters Betweenness Centrality (BC) and node degree were used to identify hub genes. The network is available as a Supplementary XML File.

## Data Availability Statement

The datasets presented in this study can be found in online repositories. The names of the repository/repositories and accession number(s) can be found below: https://www.ebi.ac.uk/metagenomics/, E-MTAB-9416 and https://www.ncbi.nlm.nih.gov/genbank/, GSE148088.

## Ethics Statement

This study was approved by the Ethics Committee and CCPPRB (Comité Consultatif de Protection des Personnes dans la Recherche Biomédicale) of Paris Cochin. All patients gave their written consent for the use of their placenta and blood samples. For Angers, the collection and use for research purpose (including genetic analyses) of placentas from pregnancies complicated with IUGR or healthy pregnancy have been approved by the Ethics Committee of Angers. The patients/participants provided their written informed consent to participate in this study.

## Author Contributions

DV, FM, and CM conceived the work and drafted it. DV performed qRT-PCR experiments. SJ performed the microarray work. GG drafted the manuscript and contributed human samples. CR and CA performed RNA and qPCR experiments and drafted the manuscript. All authors contributed to the article and approved the submitted version.

## Conflict of Interest

The authors declare that the research was conducted in the absence of any commercial or financial relationships that could be construed as a potential conflict of interest.

## Footnotes

1 http://bioinfo.vanderbilt.edu/webgestalt

2 https://www.gsea-msigdb.org/gsea/index.jsp

3 http://www.mirbase.org/

4 http://starbase.sysu.edu.cn

5 http://diana.imis.athena-innovation.gr/DianaTools/index.php?r=lncBase/index

6 https://cytoscape.org/

## Supplementary Material

Supplementary Figure 1 GSEA analysis of the data from the present paper in comparison with published data, demonstrating an extreme similarity of our data compared to published datasets (Shankar et al., 2015).

Supplementary Figure 2 Presentation of the network of deregulated genes in the predicted targets of miR-16, miR-455, and miR-365.

Supplementary Figure 3 STOX1 expression levels at the probe level along the gene. Junction probes analysis revealed that a large part of the 3′ exon is less present in FSK-streated cells. This decrease in a series of probes reveal that STOX1A is twice as abundant as STOX1B when the cells are not fused, while in the context of fusion the ratio STOX1A/STOX1B drastically changes.
