# Supplementary figures and images for: Urothelial Cancer Associated 1 (UCA1) and miR-193 Are Two Non-coding RNAs Involved in Trophoblast Fusion and Placental Diseases
Source: Front Cell Dev Biol. 2021 May 13;9:633937. doi: 10.3389/fcell.2021.633937 (PMC8155540; doi:10.3389/fcell.2021.633937)

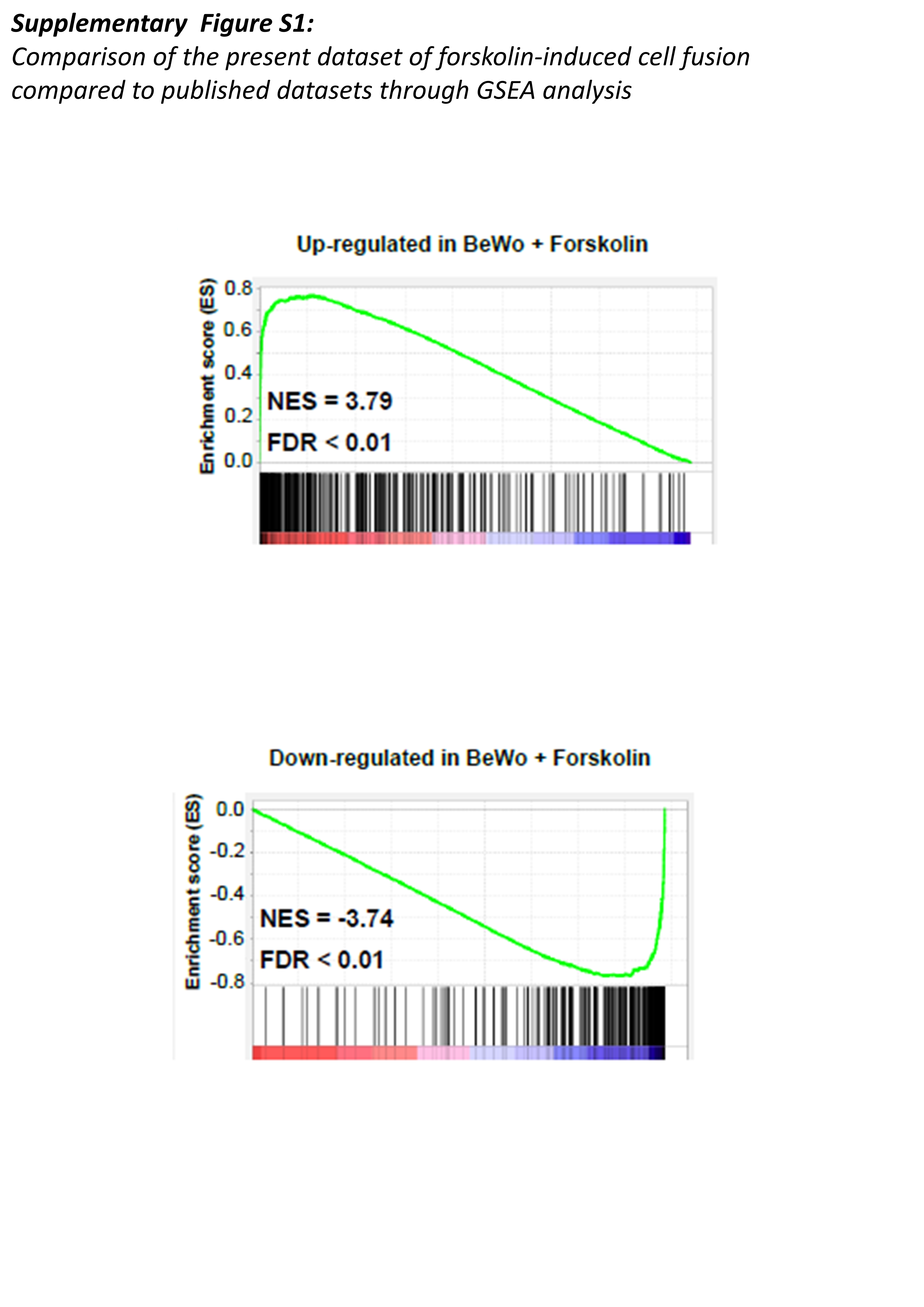

Supplement: Supplementary Figure 1 — GSEA analysis of the data from the present paper in comparison with published data, demonstrating an extreme similarity of our data compared to published datasets (Shankar et al., 2015). [file Image_1.TIF]

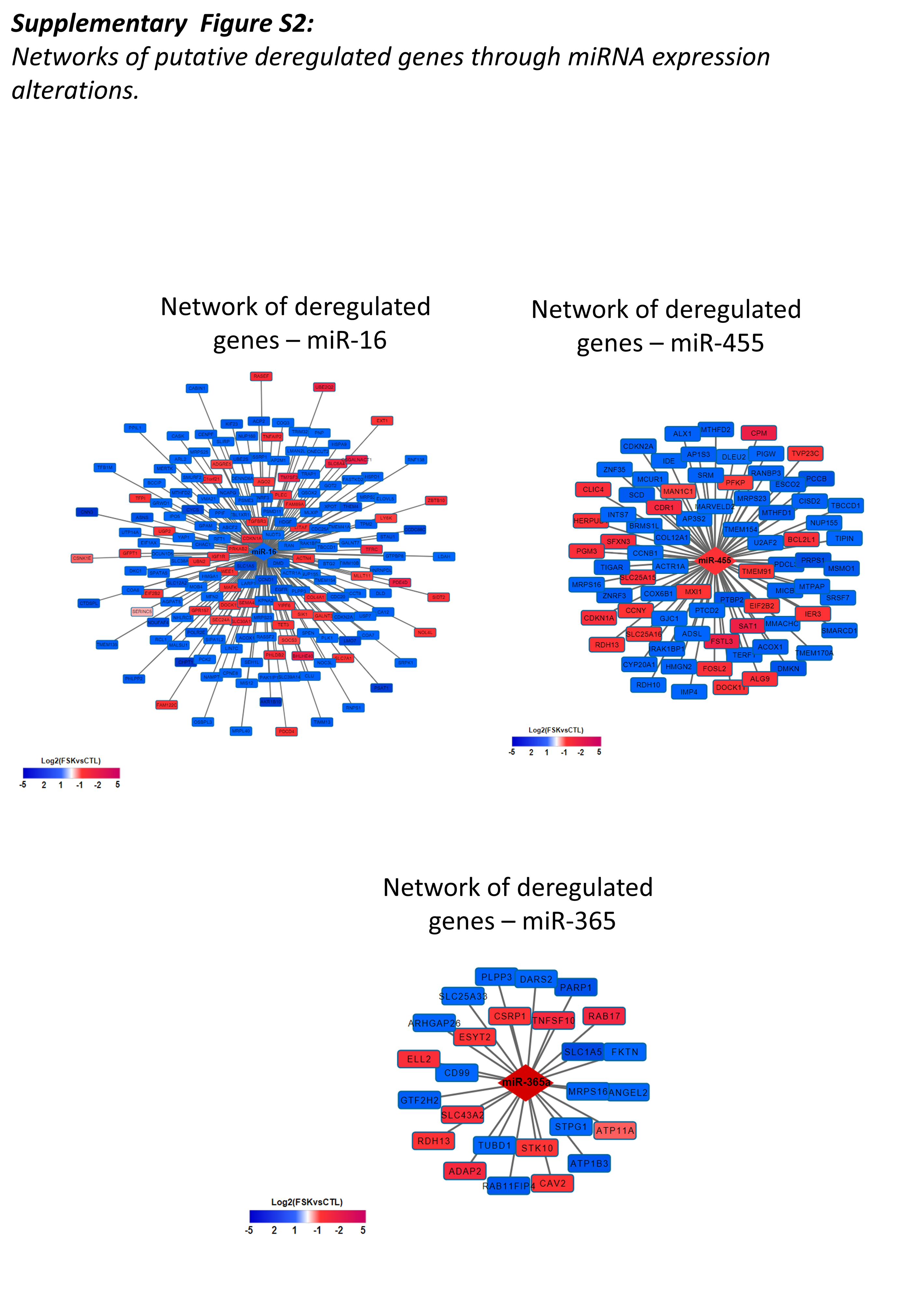

Supplement: Supplementary Figure 2 — Presentation of the network of deregulated genes in the predicted targets of miR-16, miR-455, and miR-365. [file Image_2.TIF]

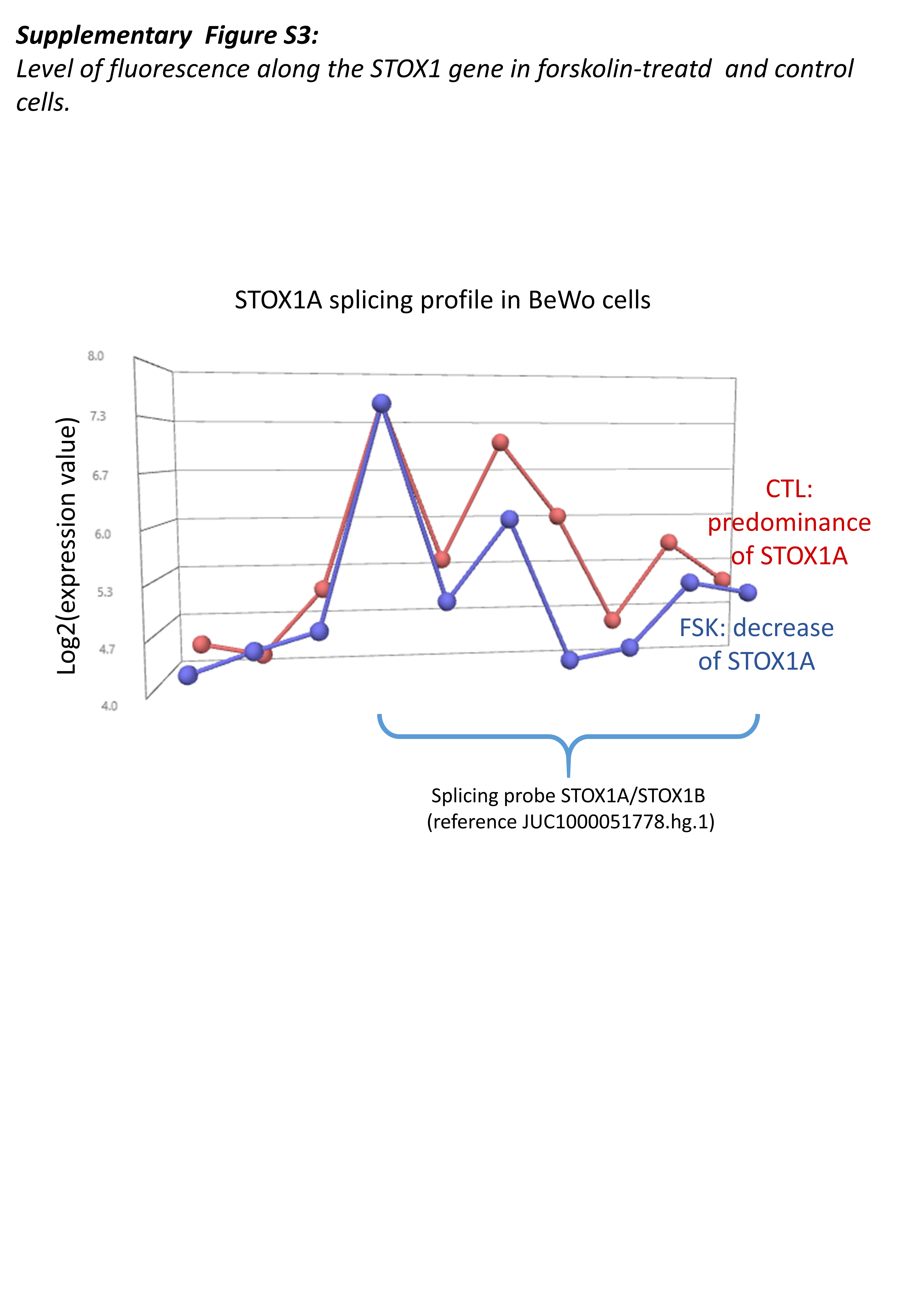

Supplement: Supplementary Figure 3 — STOX1 expression levels at the probe level along the gene. Junction probes analysis revealed that a large part of the 3′ exon is less present in FSK-streated cells. This decrease in a series of probes reveal that STOX1A is twice as abundant as STOX1B when the cells are not fused, while in the context of fusion the ratio STOX1A/STOX1B drastically changes. [file Image_3.TIF]
